# Synovial Tissue Heterogeneity in Rheumatoid Arthritis and Changes With Biologic and Targeted Synthetic Therapies to Inform Stratified Therapy

**DOI:** 10.3389/fmed.2019.00045

**Published:** 2019-03-19

**Authors:** Lylia Ouboussad, Agata N. Burska, Andrew Melville, Maya H. Buch

**Affiliations:** ^1^Leeds Institute of Rheumatic and Musculoskeletal Medicine, University of Leeds, Leeds, United Kingdom; ^2^NIHR Leeds Biomedical Research Centre, Leeds Teaching Hospitals NHS Trust, Leeds, United Kingdom

**Keywords:** rheumatoid arthritis, biologics, JAK inhibitors, synovial tissue, histology, cytokine, gene expression, pathotypes

## Abstract

The treatment of rheumatoid arthritis (RA) has been transformed with the introduction of biologic disease modifying anti-rheumatic drugs (bDMARD) and more recently, targeted synthetic DMARD (tsDMARD) therapies in the form of janus-kinase inhibitors. Nevertheless, response to these agents varies such that a trial and error approach is adopted; leading to poor patient quality of life, and long-term outcomes. There is thus an urgent need to identify effective biomarkers to guide treatment selection. A wealth of research has been invested in this field but with minimal progress. Increasingly recognized is the importance of evaluating synovial tissue, the primary site of RA, as opposed to peripheral blood-based investigation. In this mini-review, we summarize the literature supporting synovial tissue heterogeneity, the conceptual basis for stratified therapy. This includes recognition of distinct synovial pathobiological subtypes and associated molecular pathways. We also review synovial tissue studies that have been conducted to evaluate the effect of individual bDMARD and tsDMARD on the cellular and molecular characteristics, with a view to identifying tissue predictors of response. Initial observations are being brought into the clinical trial landscape with stratified biopsy trials to validate toward implementation. Furthermore, development of tissue based omics technology holds still more promise in advancing our understanding of disease processes and guiding future drug selection.

## Introduction

Rheumatoid arthritis (RA) is a complex, genetically and biologically heterogeneous autoimmune disease. It is characterized by a systemic inflammatory arthritis. The treatment of patients with RA has evolved considerably in recent years owing to the successful development and widespread use of biologic disease modifying anti-rheumatic drug (bDMARD) therapy, with more recent introduction of targeted synthetic DMARDs (tsDMARD) in the form of small molecules inhibitors. However, up to 40% patients in clinical trials fail to respond, also reflected in real-world practice; and a sizeable proportion fail to achieve the target of therapy, mainly clinical remission where appropriate or low disease activity (1, 2). Personalized medicine, i.e., tailoring therapy to individual patient (or, put simply, “choosing the right drug for the right patient”), has the potential to improve response rates, but has proven challenging to implement. If it is to be successful, the identification of reliable biomarkers will be of prime importance.

In this mini-review, we summarize the evidence for synovial tissue heterogeneity, and tissue studies that have evaluated change in cellular and molecular markers following currently available bDMARD and tsDMARD specifically that could aid treatment selection.

## The Synovium, Principal Target of Inflammation

The synovium is the principal target of inflammation in RA, undergoing marked pathological changes compared to healthy tissue. The study of RA synovial tissue has offered insights at a cellular level into multiple aspects of the disease, from identifying pathogenic processes and pathways (3, 4); to explaining clinical manifestations. Furthermore, changes in synovial tissue following successful treatment allow better understanding of mechanism of drug action (5–7).

Synovial tissue samples can be obtained via arthroscopic or ultrasound (US)-guided biopsies. The US-guided approach has been shown to be safe, with reproducible tissue quality/RNA yield (8), and has the advantage of enabling joint assessment for synovial thickness (gray-scale score) and vascularity (Power Doppler-PD), associated with active synovial inflammation (9).

## Healthy Synovium

In health, the synovial membrane contains relatively few cells, consisting of an intimal lining layer of 1–2 cell thickness and a distinct synovial sublining layer (10). The intima comprises fibroblast-like synoviocytes (FLS, also known as synovial fibroblasts or type B synoviocytes) intercalated with macrophage-like synoviocytes (MLS, also called type A synoviocytes) (11). The sub-lining layer is a well-vascularized connective tissue, containing collagen fibers and evenly dispersed FLS and MLS (11).

The synovial membrane is key to the structure and function of the healthy synovial joint. The synovial membrane controls transport to and from the synovial cavity, thus maintaining the composition of synovial fluid as well as overall joint homeostasis and integrity. The intimal lining is particularly important, as its lack of tight junctions or a true basement membrane allows the ingress and egress of various cells and proteins (12). Intimal FLS orchestrate proceedings, controlling the synovial fluid volume, secreting hyaluronan for lubrication, clearing intra-articular debris, regulating various immunological processes, and maintaining the extracellular matrix (ECM) of the sublining (13).

## RA Synovium

In RA, the synovial tissue becomes markedly expanded, with a striking increase in cellular infiltration. This leads to hallmark “pannus” formation at cartilage-bone interfaces; pannus can be composed of macrophages, FLS, leucocytes, plasma cells, and mast cells (14), and behaves like a locally invasive tumor, mediating damage and erosion formation in later disease (15). The intimal lining can expand to 10–20 cells in thickness, partly due to an increase in FLS, but mostly due to infiltration by bone marrow-derived MLS recruited from the circulation (15). Highly activated macrophages send pro-inflammatory signals to intimal FLS, inducing invasiveness, and to B cells, which in turn produce various pro-inflammatory mediators. Paracrine and autocrine signaling networks develop in this way, further propagating synovitis (16). Sub-lining MLS have been associated with disease activity (17) and synovial inflammation measured on magnetic resonance imaging (MRI) (18), and therefore appear of paramount importance to the inflammatory joint reaction (19). Proliferation of FLS are a prime cause of synovial hyperplasia, and major mediators of damage to cartilage and bone, via both direct and indirect interactions, including production of inflammatory mediators, adhesion molecules, proteolytic enzymes and pro-osteoclastogenic factors (13). T cells are able to establish important crosstalk with antibody-producing plasma cells (15, 20, 21). When present, CD3+ T cells in the RA synovium are mostly found in deeper sub-lining layers, where they may be homogeneously or randomly distributed, or clustered in follicle-like structures (19). Similarly, B cells, when present, are mostly organized in follicular structures, which can act as pro-inflammatory, immunological niches (19).

## Heterogeneity of RA Synovitis

RA synovitis is highly heterogeneous, with diverse cellular and molecular signatures (22, 23). In recent years distinct patterns have been recognized, primarily according to the composition, organization and localization of cellular infiltrates. Studies have revealed RA synovial ‘pathotypes’ (7, 24), namely, lymphoid, myeloid, pauci-immune, and fibroid variants (other patterns, such as granulomatous synovitis, have also been described). The lymphoid pathotype is characterized by lymphoid infiltrates, which may be diffuse (small, loosely arranged lymphocyte clusters) or follicular (large aggregates of lymphocytes organized in ectopic lymphoid structures). The latter may develop germinal centers containing T follicular helper (Tfh) cells highly expressing of programmed cell-death (PD-1), C-X-C chemokine receptor 5 (CXCR5), B-cell lymphoma (Bcl6), and Inducible T cell costimulator (ICOS) (7, 24, 25). Cellular composition of tissue defined as myeloid pathotype shows a less abundant B and T cells aggregates compared to the lymphoid subgroup, and presence of sublining macrophages. By contrast, the ‘pauci-immune’ (7) (or ‘low inflammatory’) pathotype shows minimal infiltrating immune cells (24). The fibroid pathotype has complete absence of aggregates and little immune infiltration comprising hyperplastic tissues.

FLS are also not a uniform population but segregate into different phenotypes based, in part, on their cytokine profiles (26). Additionally, functionally distinct disease-associated subsets of fibroblasts are recognized in RA synovium (27) including a study based on surface expression of CD34, THY1, and CDH11 (28). T and B cells infiltrating the inflamed synovium in RA show the highest degree of qualitative and quantitative heterogeneity. Whilst the relation of fibroblast subsets to clinical outcomes remains to be elucidated, these may prove to be instructive biomarkers.

## Synovial Tissue Gene Expression Profiles

Early gene expression of RA synovial tissue studies identified distinct profiles and revealed the presence of multiple activated signaling pathways (29–31). Perhaps unsurprisingly given its clinical heterogeneity, expression of molecular signatures in RA is likewise heterogeneous. Gene expression profiles can be modulated by disease activity and the burden of inflammation in synovial tissue (32). Gene expression in RA synovial (intimal) lining cells specifically has been analyzed using a laser mediated micro-dissection (LIMM) approach (33). Data analysis using clustering revealed two distinct RA subgroups associated with increased expression levels of inflammation-related genes [compared with osteoarthritis (OA) control tissue] involved in the tumor necrosis factor TNF-activated interferon regulatory factor (IRF1)- interferon (IFN)- signal transducer and activator of transcription 1 (STAT1)- pathway (34). Three molecularly distinct forms of RA tissues have also been identified by the same group; the first characterized by genes involved in inflammation and the adaptive immune response [matrix metalloproteinase (MMP) 1 and 3 genes, STAT-encoding and -induced genes and antigen-presenting-cell–related genes], the second characterized by genes involved in extracellular matrix remodeling (genes involved in degradation of cartilage and subchondral bone), and the third with a low-inflammation gene signature similar to that of osteoarthritis (30, 31). Increased receptor activator of nuclear factor kappa-B ligand (RANKL) (35) and decreased osteoprotegerin expression (36) have also been detected in actively inflamed RA synovial tissue. These findings, along with the lack of tissue repair signatures, support the hypothesis of inflammation-driven joint remodeling in RA, characterized by uncoupling of destructive and reparative processes (37). A number of transcription factor families, such as nuclear factor kB (NF-kB) and the activator protein 1 (AP-1), were established early on as chief regulators of gene expression in the inflamed synovium (38). Gene expression analysis of FLS indicates the presence of 2 subtypes, with high-inflammatory FLS expressing transforming growth factor (TGF)-β/activin A–inducible genes and FLS from low inflammatory synovial tissue predominantly expressing growth factor genes (39). Distinct molecular signatures indicating pathways relating to T cell-mediated immunity and major histocompatibility complex (MHC) class II mediated immunity (amongst others) upregulated in early RA, and pathways relating to the cell cycle upregulated in later disease (40) have been reported. Similarly differential gene expression between high and low inflammatory subsets of RA patients in relation to disease duration has been observed (29).

## Gene Expression Analysis Across Synovial Pathotypes

Differential gene expression has also been confirmed across the RA synovial pathotypes described earlier, providing further evidence for different molecular mechanisms underlying these variants. The lymphoid type is characterized by increased expression of genes associated with B cell and plasmablast activation and differentiation [including CD19, CD20, X-box binding protein XBP1, immunoglobulin heavy and light chains, CD38 and C-X-C motif chemokine ligand 13 (CXCL13)], as well as the Janus kinase JAK/STAT pathway and interleukin 17 (IL-17) signaling (24). In another study, patients with lymphoid aggregates again displayed activation of the JAK/STAT pathway, but also the IL-7 pathway, as well as genes associated with lymphoid neogenesis [such as CXCL13, C-C chemokine ligand 21 (CCL21), and receptor CCR7 and Lymphotoxin alpha (LTα)] and B-cell receptor activation, supporting the existence of a link between tertiary lymphoid structures and the local humoral response (41). In the myeloid pathotype, activation of NF-κB pathway genes (including TNFα, IL-1β, IL-1RA, intracellular adhesion molecule ICAM1, and MyD88), the inflammatory chemokines CCL2 and IL-8, and granulocyte and inflammatory macrophage lineage genes (such as S100A12, CD14, and OSCAR) were identified. In the fibroid pathotype, genes associated with fibroblast and osteoclast/osteoblast regulation were found to be involved, including fibroblast growth factor FGF2, FGF9, BMP6, and osteoprotegerin. Higher expression of Wnt and TGFβ signaling pathway components, as well as “angiogenesis module” genes, were also identified (24). The pauci-immune variant shares characteristics with the aforementioned pathotypes in terms of inflammatory response gene expression, with “M2 monocyte module” genes particularly activated (24, 42). Expression of IL-6, IL-6 receptor components (IL-6R and IL-6ST/gp130), and its associated signaling component STAT3 was broadly observed across all phenotypes, consistent with the multiple roles of the IL-6 pathway in both lymphocyte and fibroblast biology (24, 43). The existence of different gene expression profiles according to RA histological pathotype was also confirmed by Klimiuk et al. who demonstrated increased transcriptional activity of TNFα, IL-1, IFNγ, IL-10, and TGFβ in follicular synovitis, compared with diffuse synovitis (44).

Recently, a machine learning algorithm was able to predict RA synovial gene expression subtype according to 20 histological features. Three subtypes were pre-identified based on RNA-seq clustering: high inflammatory, low inflammatory, and mixed. The high inflammatory subtype showed enrichment of pathways of immunity, immune cell signaling (including SH2, SH3, JAK/STAT, and TNF-mediated signaling), immunoglobulins, chemokines, and cytokines. The low inflammatory subtype was defined by enrichment of transforming growth factor β pathways, glycoprotein synthesis, and cell adhesion genes (45). Distinct myeloid and lymphoid synovial histological subtypes were not identified, in contrast to previous studies (24), but the high inflammatory subtype displayed elevated expression of genes previously attributed to these in the literature.

## Synovial Tissue Studies to Predict Response to Biologic and Targeted Therapies

### General Synovial Tissue Biomarkers of Response To Therapy

#### CD68 Macrophage

Effective treatment can modify synovial histology, cytokine and gene expression, with ineffective treatment having little impact, thus providing a means to assess for pathological response (46). Synovial sublining (CD68) macrophage numbers and macrophage expressed cytokines have been shown to correlate with disease activity, and change in sublining macrophage to be the optimal indicator of effective therapy, thus providing a potential early predictive biomarker of drug response (6, 47, 48). A recent study demonstrated that the transcriptional profile of isolated RA synovial macrophages highlighted different subpopulations of patients and identified 6 novel transcriptional modules that were associated with disease activity and therapy (49). The authors suggest that transcriptional signatures in macrophages regardless of location (sublining vs. synovial lining) predict responsiveness to specific non-biologic and/or biologic therapies.

#### Synovial Pathotypes and Response

A study by Dennis et al. suggested myeloid and lymphoid pathoypes may predict therapeutic sucess with TNF inhibitors (TNFi) and IL-6-targeted tocilizumab, respectively (24). Analysis of serum chemokines further suggested these two pathotypes correlate with raised serum suloble intercellular adhesion molecule 1 (sICAM) and CXCL13 (sICAM/CXCL13) compared to high CXCL13/sICAM, respectively. These initial observations however have not been validated in other cohorts using the serum correlates (50) indicating the need for additional such synovial tissue studies. Nevertheless, stratifying patients by synovial pathotype may inform choice of targeted therapy.

Multiple types of therapies will be discussed in detail below, these are summarized in Table 1 together with key findings which indicate response to biologic and synthetic targeted DMARDs.

**Table 1:** Rheumatoid synovial tissue studies of biologic and targeted synthetic DMARDs.

	**Drug**	**RA Population**	**Analysis type**	**Key findings**	**References**
**ANTI CYTOKINE THERAPY**					
*IL-6 blockade*	TCZ	30 early RA (disease duration <1 year); treatment-naïve	Gene expression microarrays, IHC	Significant decrease in the expression of various chemokines and T-cell activation genes.	(51)
	TCZ	10 bDMARD treated RA patients 10 controls: RA patients on no bDMARDs	IHC	Complete blockade of IL-6. Inhibition of CD20, CD29, and JNK in MAPK implicates TCZ efficacy compared with MTX.	(52)
*TNF inhibitors*	IFX	32 RA patients	IHC	Reduction in synovial TNF expression in IFX responders and non-responders. Unchanged TNF in extreme non-responders	(53)
	IFX	143 active RA patients	IHC	Higher intimal and sub-lining TNF expression in IFX responders vs. non-responders.	(54)
	IFX	62 RA patients	IHC and gene expression arrays	Baseline whole synovial biopsy microarray unable to identify TNFi non-responders.	(55)
	ADA	25 RA patients	Global gene expression profiles arrays at T0 and T16, IHC	Poor response to ADA associated with:- Upregulation of genes from cell division and immune responses pathways in poor responders.- High baseline synovial expression of IL-7R, CXCL11, IL-18, IL-18ra), and MKI67.	(56)
	Several TNFi	86 RA patients	IHC	High synovial lymphoid neogenesis, with B and T cell aggregates, correlated with poorer clinical outcomes. Reversal of these aggregates associated with good response.	(57)
**CELL-MEDIATED THERAPY**					
*B-Cell depletion*	RTX	13 RA patients	IHC, digital image analysis, gene expression	Significant decrease synovial B cells post-RTX but not completely depleted compared to peripheral B cells. No strong correlation with clinical response.	(58)
	RTX	20 RA patients	qPCR	Responders have higher expression of macrophage and T cell genes. Non-responders showed higher expression of interferon-α and signaling genes.	(59)
	RTX	24 RA patients	IHC, flow cytometry	Significant lower infiltration of CD79^+^CD20^−^ plasma cells in the synovium associated with the reduction in peripheral blood B-cell repopulation.	(60)
	RTX	24 RA patients	IHC	Clinical response predicted by changes in cell types other than B cells, mainly number of synovial plasma cells.	(61)
	RTX	17 RA patients	IHC	RTX treatment associated with rapid decrease in synovial B cell numbers.	(62)
**T-CELL CO-STIMULATION BLOCKADE**					
	ABT	16 RA patients	IHC	Significant downregulation of pro inflammatory genes, notably IFNγ.Only specific reduction in synovial CD20^+^ B cells, in responders.	(63)
	ABT	20 RA patients(10 ABA and 10 MTX)	IHC	Increase in CD29 and ERK in MAP kinases.	(64)
**MIXED BDMARD COHORT**					
	NSAIDs and DMARDs with/without bDMARD (ADA, ETN, IFX, ANK, RTX)	49 RA patients and 29 RA	GeneChip® Human Genome U133 Plus 2.0 Arrays (Affymetrix, Inc.) ELISA, IHC	A myeloid phenotype (high serum sICAM1/low CXCL13) prevalent in responders to TNFI therapy A lymphoid pathotype (high serum CXCL13/low sICAM1) prevalent in responders to TCZ.	(24)
	TCZ, MTX, RTX	Early RA (mainly <1 year disease duration), pre- and post-3 months TCZ (*n* = 13 and 12 respectively) or MTX (*n* = 2 × 8 samples) TNFi-failure RA pre- and post 3 months RTX (*n* = 2 × 12 samples)	GeneChip Human Genome U133 Plus 2.0., Affymetrix, IHC	Over-expressed baseline tissue GADD45B and PDE4D in first-line MTX and bDMARD non- responders	(65)
**SMALL INHIBITORS (JAKi)**					
	TOFA	14 RA patients	ELISA, IHC, qPCR.	Reduced synovial mRNA expression of MMP1 and MMP3 and IFN-regulated genes. Clinical improvement correlated with reductions in STAT1 and STAT3 phosphorylation.	(66)
	TOFA	Varied/unclear	Synovial explants and tissue culture of primary RASFs, qPCR, WB, and ELISA	Decrease in metabolic functions (mitochondrial pathways, ROS production and glycolysis), indicating that the JAK-STAT signaling is a mediator between inflammation and cellular metabolism.	(67)
	Baricitinib	27 RA samples	Tissue culture experiments on FLS	Abrogation of IFNγ-stimulated FLS invasion by targeted inhibition of JAK.	(68)

*ABT, abatacept; ADA, adalimumab; ANK, anakinra; bDMARD, biologic disease modifying anti-rheumatic drug; ELISA, enzyme-linked immunosorbent assay; ERK, Extracellular signal-Regulated Kinase; ETN, etanercept; FLS, fibroblast-like synoviocytes; GADD45B, Growth Arrest And DNA Damage Inducible Beta; IHC, immunohistochemistry; IFN, interferon; IFX, infliximab; JAKi, janus kinase inhibitor; MAPK, mitogen activated protein kinase; MMP, matrix metalloproteinase; MTX, methotrexate; PDE4D, Phosphodiesterase 4D; qPCR, quantitative polymerase chain reaction; RA, rheumatoid arthritis; RTX, rituximab; SF, synovial fibroblast; STAT, signal transducers and activators of transcription; TCZ, tocilizumab; TNF, tumor necrosis factor*.

### Anti-cytokine Therapies

#### Tumor-Necrosis Factor-Inhibitors

Synovial studies have offered useful insights into the mechanism of action of TNFi. TNFi have been shown to regulate chemokine and leukocyte trafficking (69) likely explaining the reduction in the synovial cellular infiltrate observed; with reductions in synovial tissue expression of IL-6, IL-8, granulocyte macrophage colony stimulating factor (GM-CSF), macrophage chemoattractant protein-1 (MCP-1), IL-1β, TNF, and vascular endothelial growth factor (VEGF) (70).

Several studies have sought to identify predictors of response to TNF blockade through examination of synovial tissue cytokine expression. Baseline synovial TNF levels (intimal and sub-lining) predicted response to infliximab in one study (54), although another similar study did not reproduce this finding (53). Decreased sub-lining TNF expression was, however, seen in responders. A prospective study of 86 patients found higher proportions of synovial lymphoid aggregates in poor responders to treatment, despite higher rates of TNFi use. Baseline lymphoid aggregates were an independent predictor of poor response in multivariate analysis, and reversal of these histological changes was seen in over half of treatment responders (57). Addition of lymphocyte aggregates to sub-lining TNF expression (54) improved infliximab response prediction, but still only accounting for 29% variance (71); thus insufficient for clinical application. An early RA synovial gene expression study found that mRNA levels pertaining to several inflammatory pathways were associated with response to TNFi therapy, suggesting a role for synovial gene expression profiles as response predictors (72). Another study identified a number of negative predictors of response to adalimumab, another TNFi biologic, including baseline synovial expression of IL-7 receptor alpha chain (IL-7R), CXCL11, IL-18, IL-18 receptor accessory (IL-18rap), and MKI67 (63). However, a larger gene expression study using whole synovial tissue samples pre- and post-infliximab did not identify any predictors, perhaps because of the confounding presence of lymphoid aggregates (55).

#### Tocilizumab

Tocilizumab is a clinically effective humanized anti–IL-6R monoclonal antibody that inhibits membrane IL-6R– and soluble IL-6R (sIL-6R)–mediated signaling. The aforementioned study by Dennis et al. (24), suggested lymphoid pathotype as predictive of response. In another study, paired synovial tissue biopsies taken at baseline and post-treatment with tocilizumab showed a significant decrease in the expression of various chemokines and T-cell activation genes (51). When compared with gene expression data following other treatments, results showed strong correlation with methotrexate and B-cell depleting agent rituximab, but notable differences with adalimumab (51). A further study of synovial histology post-tocilizumab demonstrated a complete block of synovial IL-6 and a significant reduction of B-cells, CD29 and phospho-JNK. ERK was increased in the tocilizumab group compared to a methotrexate-treated control group, whilst TNF, MMP-3, and CD68 were similarly expressed in both groups. Therefore, inhibition of IL-6/CD20/CD29 may be differentially involved in tocilizumab efficacy compared with methotrexate (52). A more recent study in 33 early RA patients suggested higher expression of TNF-induced transcripts in early RA synovitis was associated with higher disease activity, and predicted poor response to first-line therapy (that comprised either methotrexate, tocilizumab or rituximab therapy) (65). Finally, an exploratory study by Das et al. suggested persistent synovial IL-6 mRNA expression (following rituximab inefficacy) associated with subsequent tocilizumab response (73).

### Cell Mediated Therapies

#### B-Cell Depletion: Rituximab

Treatment with the anti-CD20 monoclonal antibody rituximab significantly decreases synovial B cells, but, unlike in the periphery, does not completely eradicate them. In addition, synovial B cell depletion does not correlate strongly with clinical response in RA, suggesting the effects of rituximab on synovial B cells may be necessary but not sufficient for inducing clinical efficacy (58). A separate study of RA synovial histology pre- and post-rituximab confirmed these findings, but also examined changes in other cell populations at 4 and 16 weeks. A reduction in short-lived CD138^+^ plasma cells, possibly generated locally within the synovial membrane, was found to predict clinical response, whilst delayed reductions in T cell, intimal macrophages and lymphoid aggregates were also seen, highlighting the role of B cells in sustaining inflammation and cell recruitment (74). Another study suggested that clinical response to rituximab is associated with higher residual levels of CD79^+^CD20^−^ plasma cells in the synovium (together with persistence of circultaing ACPA+ IgM plasmablasts) (60). In addition, there is evidence that baseline synovial gene expression may be able to predict response to rituximab (and lack of response), as composite “gene scores” were found to correlate with changes in disease activity (DAS-28 score) in one study (59). Genes relating to macrophage and T cell function were activated in responders.

At a more fundamental level, B cells have shown to be central to T-cell mediated synovial inflammation. This was elegantly demonstrated by a study showing that synovial T-cell clones adoptively transferred into human leukocyte antigen (HLA)-DR-matched synovial tissues xenotransplanted into severe combine immunodeficient (SCID) mice are able to enhance local production of IFNγ, TNF, and IL-1β, but only when transplanted tissues contain B-cell follicles (75). Furthermore, treatment of synovial grafts with anti-CD20 depleting agents induces not only a decrease in B-cell density but also a disruption of the overall lymphoid architecture and reduction of cytokine expression, as well as a dramatic depletion of T cells and macrophages, in keeping with the existence of an active cell network supported by B cells.

#### T-Cell Co-stimulation Blockade (Abatacept (CTLA4-Fc))

Abatacept, a recombinant fusion protein approved for the treatment of RA, blocks T cell co-stimulation by competing with CD28 for CD80/86 on antigen presenting cells. Synovial studies of the effect of and mechanism of abatacept are relatively lacking. A study of 16 RA patients compared synovial tissue pre- and 16 weeks post-abatacept in terms of gene expression and immunohistochemistry. Amongst responders, there was notable downregulation of several pro-inflammatory mediators, particularly the T-cell-related cytokine IFNγ. However, only a specific reduction in synovial CD20^+^ B cells without significant disruption in other cell populations was observed (contrasting with the observations following anti-cytokine therapies, perhaps in keeping with the more immunomodulatory role of CTLA4) (63). Whilst effects on tertiary lymphoid structures were not analyzed, these observations suggest that disruption of T-/B-cell interactions may be critical to abatacept's mode of action. In contrast to this study, a smaller study on 5 patients treated with abatacept indicated inhibition of cell proliferation, with decreases in the expression of MMP-3, CD68, CD4, CD8, CD20, CD80, and CD86 in the synovium (64).

### Small Molecule Janus-Kinase (JAK) Inhibitors

Multiple inflammatory cytokines signal via JAK-STAT pathway. Thus, JAK/STAT signaling plays a key role in several immune mediated inflammatory diseases, including RA (76). As small molecules with intracellular targets (i.e., JAK family members), JAK inhibitors represent a novel targeted therapeutic approach in RA (77).

Tofacitinib is an oral JAK inhibitor effective for the treatment of RA (78). It is a pan-selective JAKi, blocking signaling mediated via JAK1, JAK3 and, to a lesser extent, JAK2 (79). A comparison of RA synovial tissue at pre- and 4 weeks post-treatment with tofacitinib showed no change in an overall inflammation score or levels of T cells, B cells or macrophages, but reduced expression of MMPs (MMP1 and MMP3) and interferon-regulated genes, notably CXCL10. Furthermore, clinical improvement at 4 months was found to correlate with reductions in STAT1 and STAT3 phosphorylation, indicating the importance of IFNγ and IL-6 inhibition, respectively (66). In addition, a recent metabolomics study showed that adding tofacitinib to RA synovial explants and synovial fibroblasts *in vitro* led to decreased mitochondrial pathway activity, reactive oxygen species (ROS) production and glycolysis, suggesting modulation of cellular metabolism may contribute to its therapeutic effect (67).

Baricitinib, a JAK inhibitor targeting JAK1/JAK2, is another licensed treatment for RA (80). A study specifically examining FLS activity in RA showed that baricitinib abrogates IFNγ-induced invasiveness of FLS (68), which is of importance given their key contribution to pannus formation (aggressive cell masses that destroy articular cartilage and bone), one of the hallmarks of RA synovial pathobiology (81).

## Conclusion

It is well-accepted that the considerable advances in the treatment of RA need to be accompanied by a stratified approach that mitigates against the current trial and error approach of treatment decision-making, and the associated individual patient and health-economic consequences. Significant investment in biomarker studies has failed to deliver clinically meaningful tools, with the vast majority focusing on peripheral blood-based evaluation. The emphasis on synovial tissue, the primary site of RA is intuitive, from which tissue and thus disease subtypes are emerging.

The need to pull through benchside investigation of tissue biomarkers to the bedside demands more refined and innovative stratified trial design (82). We will soon see the outcomes of such initiatives [including STRAP—Stratification of Biologic Therapies for RA by Pathobiology (ISRCTN10618686) and R4-RA—A Randomized, open labeled study in anti-TNFa inadequate responders to investigate the mechanisms for Response—Resistance to Rituximab vs. Tocilizumab in RA (ISRCTN97443826)] that will inform future tissue driven trial design. These trials and other tissue-based programmes such as the recently established NIH Accelerating Medicines Partnership (AMP) RA/SLE network will also exploit high-dimensional analyses including mass cytometry, RNA-seq of selected cell populations, and single cell RNA-seq (83). Whilst the sheer volume of data in itself presents massive challenges in the clinically meaningful interpretation, the richness of data matched with improved sophisticated analytical techniques holds the promise of being able to join the field of personalized RA targeted therapy use.

## Author Contributions

LO, AB, and AM: literature Search, write up, final approval of the manuscript; MB: conception and design of the work, write up, final approval of the manuscript.

### Conflict of Interest Statement

The authors declare that the research was conducted in the absence of any commercial or financial relationships that could be construed as a potential conflict of interest.
